# The psychosocial response to a terrorist attack at Manchester Arena, 2017: a process evaluation

**DOI:** 10.1186/s40359-021-00527-4

**Published:** 2021-02-02

**Authors:** Daniel Hind, Kate Allsopp, Prathiba Chitsabesan, Paul French

**Affiliations:** 1 School of Health and Related Research, University of Sheffield, Regent Court, 30 Regent Street, Sheffield, S1 4DA UK; 2 Complex Trauma and Resilience Research Unit, Greater Manchester Mental Health NHS Foundation Trust, Manchester Academic Health Science Centre, Manchester, UK; 3 Division of Psychology and Mental Health, School of Health Sciences, University of Manchester, Manchester Academic Health Science Centre, Manchester, UK; 4 Young People’s Mental Health Research Unit, Pennine Care NHS Foundation Trust, Manchester, UK; 5 Faculty of Health, Psychology and Social Care, Manchester Metropolitan University, Manchester, M15 6GX UK; 6 Greater Manchester Mental Health NHS Foundation Trust, Manchester, UK; 7 Institute of Psychology, Health and Society, University of Liverpool, Liverpool, UK

**Keywords:** Process evaluation, Mental health, Psychosocial response, Terrorist attack

## Abstract

**Background:**

A 2017 terrorist attack in Manchester, UK, affected large numbers of adults and young people. During the response phase (first seven weeks), a multi-sector collaborative co-ordinated a decentralised response. In the subsequent recovery phase they implemented a centralised assertive outreach programme, ‘The Resilience Hub’, to screen and refer those affected. We present a process evaluation conducted after 1 year.

**Methods:**

Case study, involving a logic modelling approach, aggregate routine data, and semi-structured interviews topic guides based on the Inter-Agency Collaboration Framework and May’s Normalisation Process Theory. Leaders from health, education and voluntary sectors (n = 21) and frontline Resilience Hub workers (n = 6) were sampled for maximum variation or theoretically, then consented and interviewed. Framework analysis of transcripts was undertaken by two researchers.

**Results:**

Devolved government, a collaborative culture, and existing clinical networks meant that, in the response phase, a collaboration was quickly established between health and education. All but one leader evaluated the response positively, although they were not involved in pre-disaster statutory planning. However, despite overwhelming positive feedback there were clear difficulties. (1) Some voluntary sector colleagues felt that it took some time for them to be involved. (2) Other VCSE organisations were accused of inappropriate, harmful use of early intervention. (3) The health sector were accused of overlooking those below the threshold for clinical treatment. (4) There was a perception that there were barriers to information sharing across organisations, which was particularly evident in relation to attempts to outreach to first responders and other professionals who may have been affected by the incident. (5) Hub workers encountered barriers to referring people who live outside of Greater Manchester. After 1 year of the recovery phase, 877 children and young people and 2375 adults had completed screening via the Resilience Hub, 79% of whom lived outside Greater Manchester.

**Conclusions:**

The psychosocial response to terrorist attacks and other contingencies should be planned and practiced before the event, including reviews of communications, protocols, data sharing procedures and workforce capacity. Further research is needed to understand how the health and voluntary sectors can best collaborate in the wake of future incidents.

## Background

### Recent mass casualty incidents

Whilst mass casualty events are uncommon, the number of transnational terrorist attacks has increased globally [1] (Table 1). Those physically present at a terror attack have a 33–39% of developing post-traumatic stress disorder (PTSD) within 1 year, with 17–29% of those close to the injured, 5–6% of emergency and recovery workers and 4% of local communities similarly affected [2]. Children are particularly vulnerable [3, 4]. The economic burden of mental health care may equal the medical costs [5] with considerable unmet need [6].

**Table 1:** Mass casualty incidents discussed in the text

Location	Date	Details
London, UK	7 July 2005	London transport system bombings [111–113]
Bardo, Tunisia	18 March 2015	Mass shooting, Bardo National Museum [79–81]
Sousse, Tunisia	26 June 2015	Mass shooting, Port El Kantaoui resort [79–81]
Paris, France	13 November 2015	Mass shootings/bombing around the city [81–83]
Brussels, Belgium	19 March 2016	Bombings, Brussels airport, Maalbeek metro station [81, 114]
Manchester, UK	22 May 2017	Manchester Arena bombing [35, 86]
London, UK	3 June 2017	Vehicle ramming/stabbing, London Bridge [85]
London, UK	14 June 2017	Grenfell Tower fire [3, 86]

### Guidance for responding to mass casualty incidents

The central theme of the literature on such events is the need for a phased response (Table 2) [7, 8]. In this paper, we follow a widely-used seven-step model for designing and implementing any psychosocial response (Table 2) [8]. The *preparedness phase* should involve multi-agency planning, training, and the development of community resilience [8]. The *response phase*, typically the first 4–6 weeks after a disaster, requires universal and selective psychosocial support based on the principles of psychological first aid [9]. Assessments identify people with unmet psychosocial and mental health needs, signpost support services, monitor distress, or refer for individualised psychological interventions as appropriate [9]. In the subsequent *recovery phase*, primary care and specialist services should identify those who are still distressed, or have developed difficulties later on [8], providing evidence-based psychological interventions [9]. Preventive and therapeutic approaches are intended to reduce long-term, complex difficulties.

**Table 2:** The strategic, seven-step model of community care

Phase	Activity [8]
Preparedness phase (pre-event)	1. Strategic planning, mitigation and preparation
	2. Public prevention programmes to develop communities
Response phase (first 4–6 weeks) and continuing	3. Universal and selective psychosocial interventions
	4. Community support and development
Recovery phase (ideally from 4–6 weeks; in this project, from 7 weeks)	5. Monitoring and signposting for people in need to welfare, health and social care services
	6. Augmented primary health and social care
	7. Specialist mental healthcare

Different recovery trajectories have been observed following single-incident trauma [10, 11]. Up to 70% of people may experience mild to moderate distress but not require formal psychological interventions, particularly if they receive adequate early support [8]. Others have a deteriorating response, with the potential to develop long-term difficulties, or an initial high stress response that may or may not improve over time [8]. Delayed distress may also be experienced [10]. First responders and members of clinical care teams may be directly or vicariously traumatised, but rarely seek help [12–15], with observed PTSD rates of 8–26% dependent on exposure and pre-incident training [16–18]. As a result of these differing trajectories, the guidance advocates a stepped care approach, screens and triages individuals [19]. Low-level interventions suffice for most survivors [20], and formal psychological interventions should only be delivered when there is clinical need [21]. The evaluation of screening models [22] particularly those aimed at children and young people [23], remains a research priority.

### The national and regional policy context

The 1991–2002 NHS reforms separated purchasers and providers to engender competition [24, 25], a policy known to make inter-agency collaboration more difficult [26] In 2015, health and social care spending was devolved to the Greater Manchester (GM) Health and Social Care Partnership (HSCP) [27], an organisation jointly run by the NHS and local government [27, 28]. The HSCP aims to integrate services by bringing together representatives of ten local authorities, 12 Clinical Commissioning Groups (CCGs) [29, 30], 15 NHS care provider organisations and NHS England (NHSE)—the body which oversees NHS budget, planning, commissioning and delivery from 2013 [31, 32]. Another important regional organisation was the Strategic Clinical Network (SCN), set up by NHS England (NHSE) to provide clinical leadership to improve health and care services [33, 34].

### Overview of the psychosocial response to the Arena bombing

On 22 May 2017, a suicide bomber detonated an improvised explosive device in the foyer of Manchester Arena after a concert, killing 22 people and himself, and physically injuring 239 children and adults [35]. No preparation had been conducted for the mental health response to such a contingency (*planning phase*). In the *response* and *recovery* phases, the approach was: (a) *universal;* involving public health messages to reach anyone vicariously traumatised; (b) *targeted*; approaching those known to be directly affected; and (c) *phased*; recognising different communication and treatment needs across phases, with some survivors requiring long-term support [8, 36, 37].

In the *response phase*, a multi-sector collaboration shared *universal* messages (Table 2, Step 3). These included normalisation of distressing symptoms (such as shock, intrusive thoughts, sleep problems, etc.) [38, 39], and encouraged appropriate help-seeking. The information advised against non-evidence-based early therapy or ‘debriefing’, which is known to cause harm. Early on, some people who had been directly or vicariously affected by the incident were locally assessed and referred for specialist treatment according to risk and clinical need and on a non-systematic basis. Community support (Step 4) was provided through consultation with local schools, colleges, the media and group events, including psychoeducation and information about support on offer. Social cohesion was emphasised, to encourage mutual support and prevent reactive hate crime.

The ‘Manchester Resilience Hub’, a collaboration between four NHS mental health trusts in GM, was was set up in response to the Arena attack, during the *recovery phase.* Its overarching aim was—and remains—to reduce distress and minimise development of mental health difficulties, including post-traumatic sympotms, in the wake of the incident. The hub involves an assertive screen-and-refer outreach model [40], to systematically screen people of all ages, across the UK and beyond, with a stepped-care approach, tailoring treatment pathways to the needs of different individuals and groups [20, 41]. Those in need were initially identified by an email sent to concert ticket buyers, and are still referred, via promotion of the screening programme though traditional and social media, as well as approaches to professionals through occupational health departments (see below, Results | Implementation actions). At the hub, clinicians use an online screening tool incorporating online psychological measures, completed upon registration with the Hub, supplemented by telephone contact to assess need and triage [21, 42]. Invitations to repeat the screening are sent every 3 months.

Adult measures include the Trauma Screening Questionnaire [43], Generalised Anxiety Disorder 7 [44], Patient Health Questionnaire [45], and the Work and Social Adjustment Scale [46]. Children and Young People’s (CYP) measures include the Children’s Impact of Event scale [47], and subscales of the Revised Children’s Anxiety and Depression Scale [48] for depression, generalised anxiety disorder and separation anxiety. Established clinical thresholds are used to triage respondents; the most severe score is given priority where there is disagreement across measures. Adults at low risk are given normalisation messages and advice; those with moderate distress are encouraged to self-refer to their local ‘Improving Access to Psychological Therapies’ (IAPT) service, for brief, evidence-based, psychological interventions [49]. Adults with high levels of distress and all CYP are contacted by telephone, and referred to CYP or adult mental health services as appropriate. The Hub clinical team consists of degree-level recovery workers, who have received brief training around trauma and clinical records systems, and senior clinicians. Senior clinicians are clinical psychologists or therapists experienced in CBT or EMDR, and typically seconded from NHS mental health trusts around GM.

### Objectives and theoretical perspectives

A clinical outcomes evaluation is presented elsewhere [50]. The objectives of this process evaluation were:

- A logic model describing the resources and planning actions, necessary to implement parts of the seven-step model;
- A process evaluation relating procedures [51] and context [52] to programmatic outcomes (numbers screened and in receipt of support);
- An evaluation of how well Hub practices were embedded and sustained, using Normalisation Process Theory (NPT) [53, 54], a sociological theory of the middle range [55, 56]. This sociological concept of ‘normalisation’ should not be confused with the highly relevant psychiatric concept of ‘normalisation’, also discussed in the text, which refers to understanding intrusive and distressing thoughts as a natural part of cognitive processing while recovering from a trauma [38, 39].

## Methods

### Study design

Holistic single-case design with the unit of analysis at the level of the programme [57]. A Consolidated criteria for Reporting Qualitative studies (COREQ) checklist is provided as Additional file 1.

### Development of programme theory

**Fig. 1:**
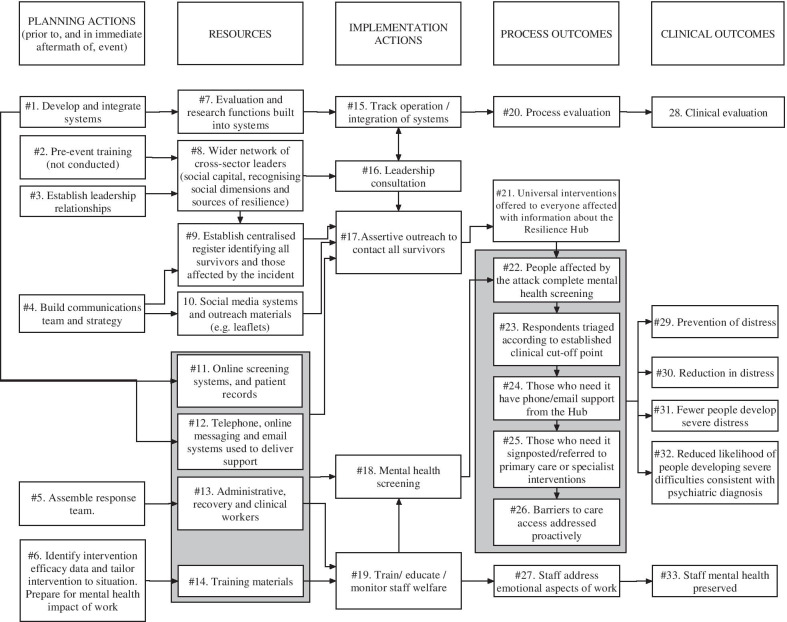
Idealised logic model for the wider collaboration. In the case study, the absence of a planning phase meant that planning actions were completed during the response phase. *RAG* Red-Amber-Green

During the planning of the psychosocial response and its evaluation, a programme theory, expressing pathways essential for its success [58–60], was developed and revised through literature review, articulation of mental models, and interviews [61]. A logic model (Fig. 1), was drafted to express the programme theory in diagrammatic form [62, 63], with planning actions, resources and implementation actions based on Flynn's Leadership in Disasters framework [7]. The grey shaded areas represent Resilience Hub-specific activities in the recovery phase (equivalent to Table 2, Step 5).

### Selection and withdrawal of evaluation participants

Key informants were drawn from the public sector (NHS, Education) and the Voluntary, Community and Social Enterprise (VCSE) sector (specialist charities dealing with mental health or support of those affected by crime and terrorism). We sampled those involved in set up and planning (‘leaders’) and frontline workers (involved in implementation) for maximum variation [64] based on organisation and programme role. Further participants were sampled theoretically [65] based on information arising from the initial interviews. This included the use of snowball sampling [66] to confirm discrepant or divergent views [67].

Participants were directly invited, by telephone, e-mail, or face-to-face, sent the information sheet, consent form and Resilience Hub logic model. Four leaders expressed willingness to be interviewed, but were unavailable during the evaluation period. Another declined on the basis of not being closely enough involved. The final sample (Table 3) comprised 21 leaders and six frontline Hub workers (n = 6).

**Table 3:** Interviewees

Role	Organisation
National expert in trauma in young people	Academia
Lead commissioner	GM health and social care
Lead for strategy and system development	GM health and social care
Clinical lead, trauma service	NHS mental health trust
Mental health lead (children and young people)	NHS mental health trust
Director of operations (CAMHS)	NHS mental health trust
Service co-ordinator, trauma service	NHS mental health trust
Medical director	NHS mental health trust
Director of Nursing and Governance	NHS mental health trust
Professional and clinical lead	NHS mental health trust
Director of psychological services (CAMHS)	NHS mental health trust
Operational manager, mental health service	NHS mental health trust
Mental health lead (adults)	NHS mental health trust/Strategic clinical network
Associate director	Strategic clinical network
Mental health lead (children and young people)	Strategic clinical network
Quality improvement manager (CAMHS)	Strategic clinical network
Clinical lead	Strategic clinical network
National lead	Third section organisation
CEO	Third sector organisation
CEO	Third sector organisation
CEO	Third sector organisation
Recovery worker	Manchester Resilience Hub
Administration and project management	Manchester Resilience Hub
Senior clinician (adults)	Manchester Resilience Hub
Senior clinician (adults)	Manchester Resilience Hub
Senior clinician (children and young people)	Manchester Resilience Hub
Senior clinician (children and young people)	Manchester Resilience Hub

### Procedures

For leaders, DH conducted consent and interviews by telephone; for frontline staff, KA conducted these processes face-to-face or by telephone. Bespoke interview guides (Additional file 2) were developed for this study. Questions for leaders were based on a conceptual framework [55, 56] for ordering the actions and roles of leaders in disasters [7] and a synthetic framework summarising published theories of how organisations successfully collaborate [68]. Leaders were also asked to give feedback on the logic model. The topic guide for frontline workers contained questions based on NPT [53] and an abbreviated cognitive task analysis [69]. Interviews, which took a median of 69 (38–107) minutes, were digitally recorded on an encrypted machine and fully transcribed. Field notes were taken during and after interviews as required.

**Fig. 2:**
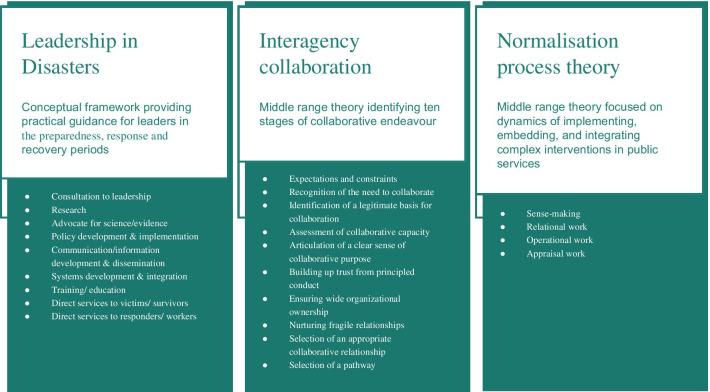
Conceptual frameworks used in the study

We used NVivo 11 (QSR International), to support a National Centre for Social Research ‘Framework’ approach to analysis [70]. DH and KA undertook all stages of the analysis of transcripts: familiarisation; identifying a thematic framework; indexing; charting; and, mapping and interpretation. Interviews were coded to the conceptual/theoretical frameworks that informed the relevant topic guides (Fig. 2), with one leader interview—which had a particular bearing on implementation—also coded within NPT [53]. Sample quotes coded to each construct of the conceptual/theoretical frameworks can be found in Additional file 3. DH and KA coded a sample of the transcripts, before conferring with other authors that the interpretations were plausible. In the results, logic model pathways are used to structure the responses of leaders which mainly related to the response stage. Minutes of meetings were consulted to enhance our understanding of the process and, where we found the over-lapping subject matter, we cross-referenced our findings with those of the Kerslake Report on the wider response to the incident [35].

## Results

**Fig. 3:**
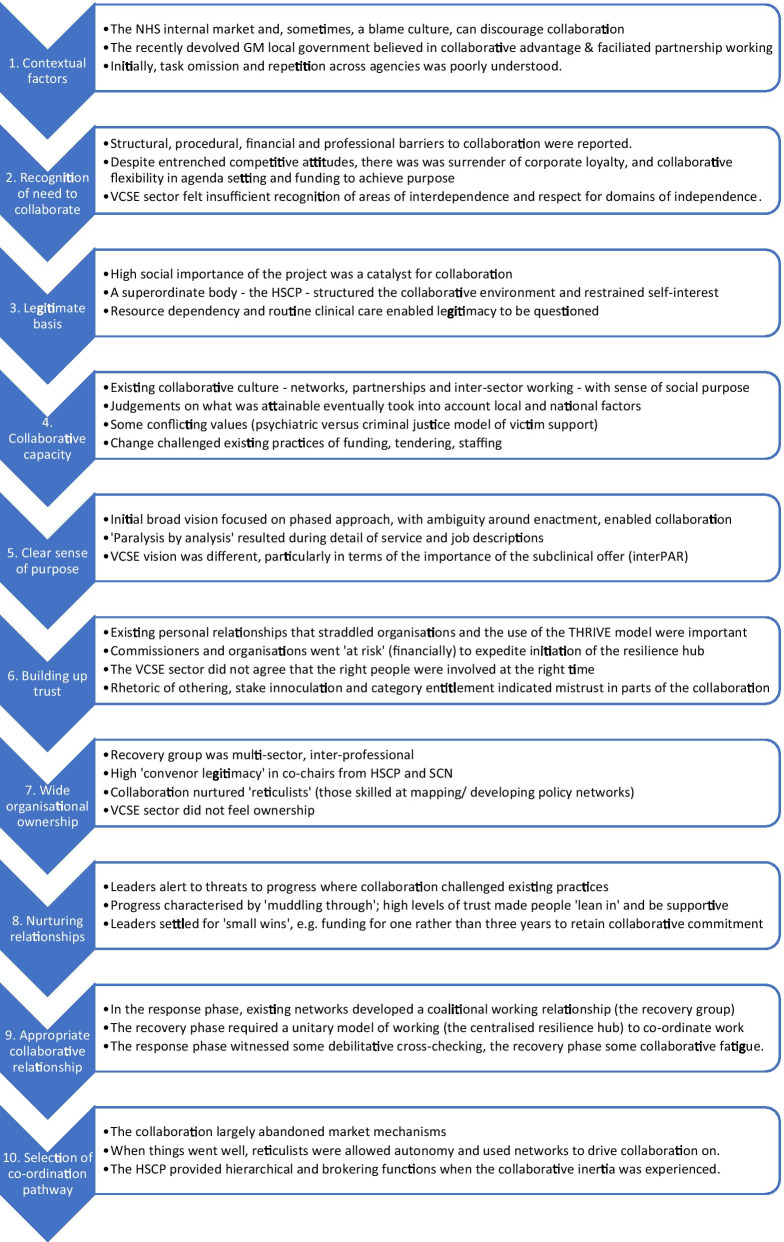
Findings based on the Inter-Agency Collaboration Framework

We cross-refer to critical pathways on the logic model (Fig. 1) using hash (#) and arrow symbols. The logic model is idealised and simplified, including one element which should be undertaken, but was not (#2), and elements which were not in place until the *recovery phase* (#11–#14, #19, #22–#25). Findings associated with the Inter-Agency Collaboration Framework are detailed in Fig. 3; further explanation of the terms used in Fig. 3, and illustrative quotes, are given in Table 4.

**Table 4:** Explanations for findings in the Inter-agency Collaboration Framework [68] (shown in Fig. 3)

Concept	Definition	Sample quote
Task omission (3.1)	Activities which are important to the collaborative objectives are not carried out [73]	“there’s been no support I’ve been all on my own’, many, many people say that from beyond Greater Manchester” (S05-F01)
Task repetition (3.1)	Organisations separately carry out actions which need only be done by one [73]	“trying to work through where we are duplicating work again and again” (S04-F03)
Appreciation of interdependence (3.2)	Organisations have similar goals but operate with different functions in different sectors [115, 116]	“the voluntary sector work, even… some of the most clinicians I respect the most… think of it as being non-clinical… not as experts … so there was a lot of people that could have been plugged into really good local, targeted voluntary sector organisations” (S12-F01)
Domains of independence (3.2)	Organisations define activities as their specialist domain [115, 116]	“we’re not just another charity you are that person that organisation fulfilling that statutory duty and that statutory duty should dovetail with the clinical mental health support and that does need to be recognised … It was a very difficult to get any acknowledgment of the existence of ourselves as an organisation with that expertise” (S05-F01)
Resource dependency (3.3)	Service delivery interactions are fundamentally dependent upon acquiring resource [105]	“there was confusion with the centre around what … money we would need and where it would come through” (S03-F01)
The fulfilment of routine clinical care (3.3)	Officials may be reluctant to permit new work which interferes with the delivery of existing programmes	“that was tricky… giving up psychological therapy staff… there are organisational commitments to which they are attached and IAPT targets” (S05-F06)
Change challenged existing practices of funding, tendering, staffing (3.4)	Culture can influence how strategy is made [117]. Change can challenge existing practices and values [118]	I don’t feel I need to worry about competitions… my job becomes one of finding the legal and other mechanisms to allow people to cooperate and work together… if I can get people to cooperate why would I waste my time [with] a convoluted procurement programme? (S03-F01) We had to absolutely get staff in and second staff from other organisations… the whole… system that we are involved in doesn’t allow that to happen (S05-F06)
Initial broad vision… with ambiguity around enactment, enabled collaboration (3.5)	A broad vision generates more momentum than blueprints. Ambiguity can make negotiation easier, serving as “the grease that allows decision-makers to co-operate” (p36 [119])	The main goal… we all agreed… was… minimising long-term… psychological difficulties… how we achieve it… was… flexible… I’m not so sure anyone… explicitly said ‘this is what we’d like you to do’. (S01-F01)
The response phase witnessed some debilitative cross-checking (3.9)	“Sooner or later, someone or some organisation, will be offended either by the actions of another organisation or by what a core group has committed it to. On the other hand, paying serious attention to accountability can be almost as debilitating, because it implies a need for a continual process of checking in both directions” (p8 [120])	the pathways document… that was the first… real test of collaboration… everybody was wanting to just sort of tweak… odd words and nuances… somebody should have said… stop, let’s just get it out… Whereas… we were all… still trying to be a bit too polite (S02/F03) We thought we’d made a decision but it then had to be sense checked by somebody else who said yes but it had to go through them and we didn’t quite understand why … at the time it felt interminable (S01/F01)
The HSCP provided hierarchical and brokering functions when the collaborative inertia was experienced (3.10)	A hierarchical model can apply where organisations with common ties are asked to work jointly in a way that would not usually occur on a voluntary basis [121]. This approach may depend on an executive body using its “position in the flow of resources to specify the nature of programmes and linkages at subordinate levels” [68]	there’s… inevitably some competition… some… sense of… whose view is best when there are differing views… there was… a little bit of… tension between providers… a bit of reverting to type… that was brokered… by the partnership and by the commissioners (S02/F03)

### Context

(Fig. 3.1). A collaborative spirit deriving from the HSCP (Fig. 3.4, 8) was evident in the close working relationships with counterparts at other organisations, the ability to ‘learn by doing’ [68] or ‘muddle through’ [71, 72] (Fig. 3.8), and the “muscle memory” (S03/F02) of partnership working, in pursuit of collaborative advantage [73, 74]. Commissioners were ambivalent about market mechanisms:

I don’t feel I need to worry about competitions… my job becomes one of finding the legal and other mechanisms to allow people to cooperate and work together (S03-F01).

**Fig. 4:**
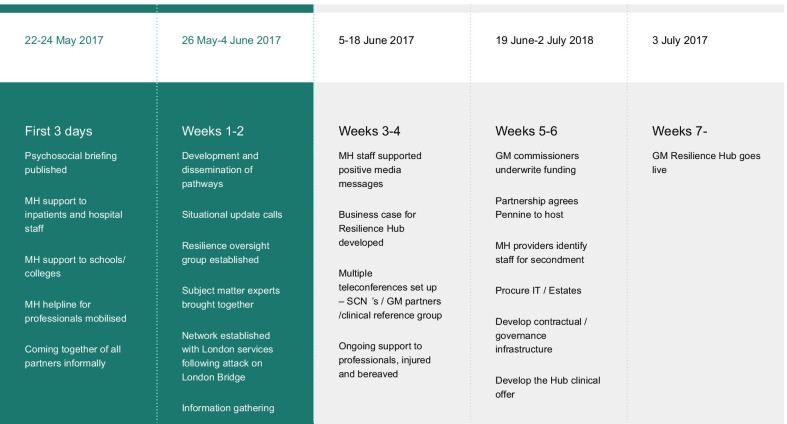
Timeline (response phase)

The perceived status and legitimacy of leaders from existing networks, the HSCP and SCN, were critical in integrating the psychosocial response in the *response phase* (Fig. 4), in the absence of pre-incident planning (Fig. 3.3, 7). Under the UK Civil Contingencies Act, 2004, statutory responder services are obliged to conduct contingency planning through Local Resilience Forums (LRFs) [75]. GM LRF had not developed mental health response systems and mental health service providers were not included in pre-event simulations (Fig. 1 #2):

What was really clear immediately to me was that we should have been involved in the start with ‘Gold Command’ and that there should have been a pre-agreed plan…something really important about having a regular update, in anticipation of major incidents of where you’ve got capacity and how you can draw that in quickly (S02-F06).

### Planning and resources

#### Partnership working

**Fig. 5:**
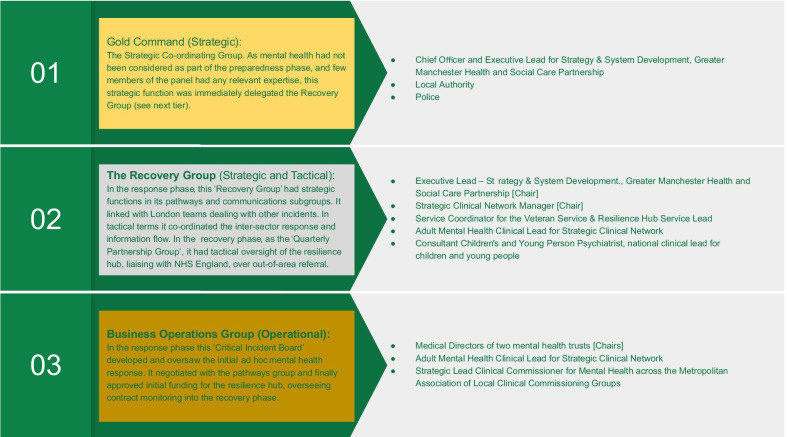
Three committees involved in the response phase

An overall strategic co-ordinating group (Fig. 5) was attended by leaders from relevant agencies (health, criminal justice, etc.). At 07:30 on the day after the incident, one member of this group—the HSCP’s Executive Lead for Strategy and System Development—was asked to convene a ‘Recovery Group’ (Fig. 5) to integrate the psychosocial response to the incident (Fig. 1, #1 ↔ #3 → #8 → #16). Group membership was rapidly extended to health, education and VCSE contexts; a national trauma expert and a NHSE representative also attended. Guided by a whole-systems approach to supporting mental health difficulties [76], the Recovery Group attempted to harness a ‘network’ or ‘system’ to increase community and individual-level resilience during the *response phase* (Fig. 4). Services were fragmented on geographic and specialist (adult/CYPMH) lines with no single point of entry. So, leaders agreed that, during the *recovery phase*, they needed a more systematic, “robust way of screening… [and] assertively outreaching people… something that no other service was commissioned to do” (S02/F01; Fig. 1, #17/#18).

we knew that our job would be to identify the people who needed help and make sure that their local NHS services or other relevant services were able to deliver that help and be able to… help people navigate through the mental health system (S02/F04).

The Recovery Group convened task-and-finish subgroups on communication workforce and clinical pathways (Fig. 1, #4, #5, #6,). Psychoeducation and informative content were rapidly tailored into factsheets and communications strategies, ensuring that messages were evidence-based and effectively worded [77] (Fig. 1, #4 → #10). Based on the guidance of experts in the field, national guidance from 2006 [36] was modified to allow highly targeted, evidence-based early intervention [78] (Fig. 1, #6,). Published and unpublished data from different mass casualty incidents were considered (Table 1, [3, 79–86]), especially those from Omagh [87, 88], because of the number of children involved and the length of follow-up.

#### The development of the Resilience Hub

An NHS trust director of operations for CYPMH, a consultant CYP psychiatrist, and the SCN Adult Mental Health Lead drafted clinical pathways [89, 90] for adults and CYP, harmonised to allow for a family-oriented approach and avoid a ‘postcode lottery’. They proposed that all local mental health care providers would provide trauma therapy-trained staff to a telephone/email-based screening/outreach programme (the ‘Resilience Hub’). Part-time secondments would preserve capacity in, and disseminate staff learning across, the system. An NHS trust Clinical and Professional Lead for Psychological Therapies and a consultant CYP psychiatrist identified bank IAPT staff trained in EMDR, trauma-focussed CBT and family therapy (Fig. 1, #5 → #13). An NHS trust Director of Operations for CYPMH and the SCN Mental Health Lead submitted a business case for the Resilience Hub to commissioners on a third committee, the Critical Incident Board (Fig. 5), covering the costs of workforce, training, infrastructure, screening, communications (Fig. 1, #11-#14, #17-#19). Following the development of the business case the Critical Incident Board approved the pathways after revisions, and handled contracting. GM CCGs and the HSCP agreed to underwrite £2.3 m for 3 years of screening and active support from June 2017 until funds from central government could be secured. The speed of decision making from the local system was crucial in enabling the hub to mobilise and commence screening at the 3 month time point. The activity was linked to an extension of an existing contract between the provider trust and one of their commissioners, who took on contract/performance reviews.

The HSCP approached the ticketing company for the names, addresses and e-mail addresses of those who had bought the 20,000 tickets (#8 → #9), for assertive outreach use by the Hub (#9 → #17). Precise specification of the purposes of use meant it took Caldicott guardians—senior individuals responsible for protecting the confidentiality of identifiable health and care data in the NHS [91]—and others seven weeks to finalise the data-sharing agreement.

The Service Co-ordinator and a consultant clinical psychologist from the veteran’s mental health service and a CAMHS Operations Director led the set-up of the Hub (Fig. 1, #1 → #11/#12). A room with appropriate cabling was secured. The ‘Patient Case Management Information System’ (PCMIS, University of York, York), already in use by the veteran’s service, was modified to capture screening data and support triage (#18 → #22 → #23).

#### Criticisms of the partnership working

**Fig. 6:**
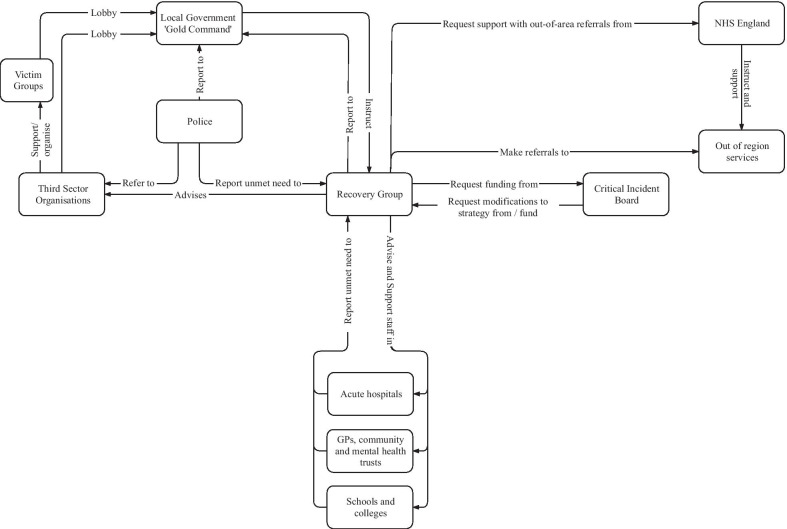
Simplified information flow within the response system. Information flow within the response system prior to setup of the Resilience Hub (the majority of listed organisations also communicated information to the public)

**Fig. 7:**
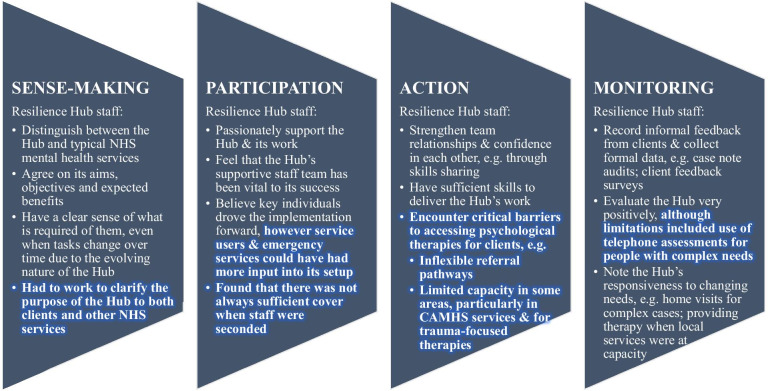
Findings based on normalisation process theory

The majority of VCSE leaders believed that the Recovery Group duplicated existing work and involved them insufficiently and brought them in too late to affect the design work. All of the VCSE leaders felt that the Recovery Group failed to understand their sector’s assets (Fig. 1, #1 ↔ #3 → #8 → #16; Fig. 3.1, 2, 6, 7). The majority believed the central offer “failed to take into account people with subclinical need” (S05/F01) in line with Ministry of Justice guidance [92]: the Recovery Group decided they did not have the evidence or resources to deliver something like the *International Program for Promoting Adjustment and Resilience* (interPAR) model [93] (Fig. 3.5).

### Implementation actions

#### Partnership working

The Recovery Group held teleconferences, initially daily then weekly, often with over fifty attendees. Delegates provided situation reports from, and disseminated information out to, health, education and VCSE service contexts (Fig. 1, #15 ↔ #16; Fig. 6). They developed a register of those affected (Fig. 1, #8 → #9), including those supported by:

- hospitals and emergency services;
- police family liaison officers or bereavement counsellors,;
- Rapid Assessment, Interface and Discharge (RAID) teams, specialist mental health services working in acute hospitals [94];
- college counsellors; or,
- charities/family liaison officers.

#### Outreach

Specialist mental health providers offered support to patients, families and professionals at major acute hospitals in receipt of the injured (#25). In interviews, it was reported that health professionals caring for the wounded had no systematic support as they came off-shift. NHS occupational health departments would not allow mental health services to contact staff directly which “compromised” (S02/F03) the fidelity of the proposed care model; NHS staff were described as often “very wary of their own occupational health department” (S02-F06). Employers and RAID teams made ‘backdoor’ referrals but some interviewees felt that ad hoc support systems, such as a drop-in centre and help-line, were not well used; affected professionals tended to “minimise” and resort to presenteeism, possibly due to cultural factors including stigma [12–15].

Phased self-help information was sent out (#10 → #17), two and six weeks after the incident, through traditional media, social media, websites and schools. Statutory sector interviewees reported difficulty getting messages out ‘intact’ outside of the region and were concerned that the media’s coverage might be counter-productive [95–97]. Information for health professionals was disseminated via splash screens, pop-up software windows, to local NHS staff via their intranet.

In the absence of ticketing data, the Recovery Group was initially unaware that the majority of those affected were living outside of Manchester (pp. 111–112, [35]). The VCSE sector and family liaison officers supported some of the underserved into sharing experiences on a social networking service, and protesting at the absence of support (Fig. 6). Recovery Group members went to great lengths to, “get an equity of response outside of Manchester” (S02-F06) including asking companies who provided travel to the concert to fund private therapy (Fig. 1, #26). NHSE teleconferenced with strategic clinical networks in other areas of the country to address barriers to care and disseminate advice/materials (#16 → #17).

#### Training

With particular expertise in blast injury trauma, the Veteran’s Service played an immediate role in educating local clinical networks. Trauma-focused therapists and accredited supervisors were in short supply, so bespoke training workshops were arranged for CBT and EMDR trauma therapists (#6 → #14 → #19). Oversubscribed, the sessions were recorded and hosted by the Psychological Professions Network on a password protected webpage. At the time of writing, mental health providers were considering accessing training on the delivery of Schwartz Rounds [98–100] to help staff address the emotional aspects of work and preserve staff mental health (#19 → #27).

#### Inappropriate care from outside of the system

All statutory sector interviewees expressed concern about the inappropriate, early use of active therapies [101–103] (#15 ↔ #16). They felt their normalisation messages, such as “it’s okay not to feel okay”, calmed the impulse to ‘just do something’ [104] amongst health workers. They reported challenging unregulated groups from overseas (pp. 48, 111, 120 [35]) who had re-traumatised people through inappropriate early intervention:

I went to a meeting… to …bring together voluntary sector groups …every time we sat at a table with this particular group they got up and left, so they wouldn’t be challenged by us… some of those people [treated by the VCSE group] subsequently have come to the Hub and been quite damaged by what they were offered… (S02-F06).

### Process outcomes

#### Reach

Systematic process outcome data collection was only undertaken as part of the Resilience Hub. There is disagreement on why the launch of the Resilience Hub was delayed, but there was late consideration of model’s appropriateness by senior civil servants, locally and nationally. The delay prevented a planned six-week mailout. Before the 3-month mailout, Hub procedures were piloted with first responders and some of the individuals who had protested at poor service access. At 31/07/2018, 1 year after the first mailout, over 7000 emails had been sent, inviting ticket purchasers and those referred from partner organisations to complete screening (#9 → #17 → #21). Of these, 3281 had completed screening (#22), 602 (18.3%) aged 0–15 years on that date, 275 (8.4%) aged 16–17 years and 2375 (72.4%) aged 18 years or over. At 31/07/2018, 79% of the individuals supported by the Resilience Hub lived outside of Greater Manchester. At 10/05/19, 66% of Hub clients had received individual phone and/or email support (#24). Table 5 illustrates the proportions of adults and CYP who had clinically significant screening questionnaire scores upon registration with the Hub. Hub staff, other clinicians, the police and VCSE workers also ran a series of targeted events for CYP, adults, and families, focused on normalising trauma responses, impact on relationships, connecting with those who have similar experiences, posttraumatic growth and resilience-building (#25).

**Table 5:** Proportion of adults and CYP at baseline with clinically significant mental health questionnaire scores, comparing Hub clients who registered within 3, 6, and 9 months of the attack

	First screen at 3 months (%)	First screen at 6 months (%)	First screen at 9 months (%)
*Adults—Baseline*			
PHQ-9	34.50	50.00	49.80
GAD-7	36.60	49.80	55.80
WSAS	41.50	61.20	58.40
TSQ	51.10	67.50	68.20
*CYP—Baseline*			
CRIES-8	84.20	82.90	92.90
RCADS Depression	13.00	21.00	17.40
RCADS GAD	19.90	23.10	35.30
RCADS Parent GAD	35.00	44.10	50.80
RCADS Parent Separation Anxiety	33.70	52.90	45.20

PHQ-9: % with scores of 10 or more, indicating moderate to severe depression [45]

GAD-7: % with scores of 10 or more, indicating moderate to severe anxiety [122]

WSAS: % with scores of 11 or more, indicating significant to severe functional impairment [46]

TSQ: % with scores of 6 or more, indicating possible PTSD [43]

CRIES-8: % with scores of 17 or more, indicating possible PTSD [123]

Each RCADs scale scored according to child’s age and gender. % with T-scores of 70 or higher, indicating scores above the clinical threshold [124]

The information in this table is adapted from [125]

#### Governance

The Recovery Group continued as quarterly Resilience Hub Partnership Board, with a remit of: developing the Hub’s role; sharing intelligence on those affected; gauging pressures on staff wellbeing; resource use; giving voices to service users and stakeholders; building an evidence base and reporting mechanism. One VCSE interviewee characterised data presentations at the Partnership Board as “opaque”:

“mainly about … how many people have filled in the questionnaire and it’s really hard to work out how many people have actually had how much one-to-one support” (S05/F01).

#### Leader evaluation

The psychosocial response inevitably involved reconfiguration of scarce resources and tensions in responding to a surge in demand (Fig. 3.2, 3):

the message I sent out… was you will prioritise these folk because there is an evidence base whereby they are more vulnerable… I’m not overriding NHS rules about clinical priority what I was doing was on the base of clinical need (S03-F01).

a young girl was due to go to the concert… couldn’t make it because of an anxiety disorder, her friends went to the concert… ended up with CAMHS appointments… (S11/F01).

knowing what capacity we’ve got in the system … and how can it be freed, whilst also ensuring that your core business happens day to day because there was a backlash – minor – but there were some people who felt that this was this was taking staff away from basic core business (S02-F06).

when we second these staff into the Hub… it was difficult because… there’s a huge amount of pressure from… GM [for] hitting targets in IAPT.

As we have noted, VCSE sector interviewees all had criticisms of the programme, although two balanced this with praise: “everybody has … done an incredibly amazing job considering the size and scale of incident” (S11/F01). Statutory sector interviewees were overwhelmingly positive about the collaboration. All stressed (Fig. 3) how existing “system relationships” (S04-F02), and the social importance of the work [105] meant that people “leant in” (S09/F01). Any “reverting to type” (S02/F03)—for instance competition over ownership of work—was swiftly brokered by the “was brokered… by the partnership and by the commissioners” (S02/F03).

we were brought together because of the severity of the incident… and we managed to put aside our vested interest… by not collaborating you would just… allow a system to maintain its cracks through which people will fall. (S05-F06).

### Interviews with Resilience Hub workers

#### Coherence: did the intervention make sense and ‘fit’?

**Fig. 8:**
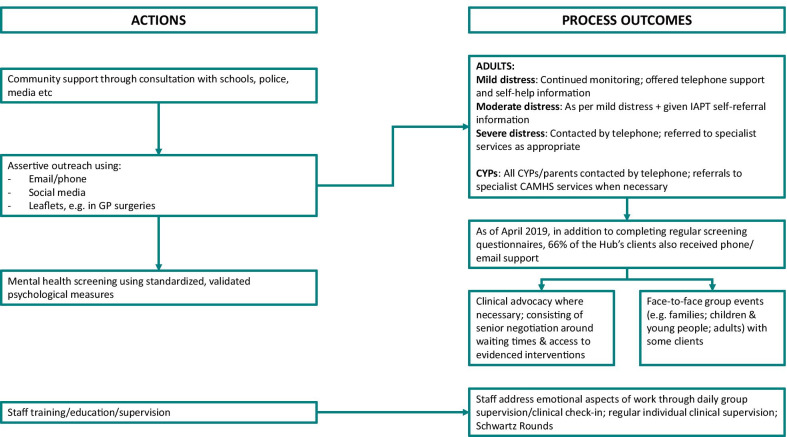
Logic model for Resilience Hub workers

Findings based on the NPT are summarised in Fig. 7; a Resilience-Hub-level logic model is provided in Fig. 8. Hub staff distinguished the Hub from other NHS services as “an all age service” with “a real focus on families that is aspirational in other services” (S02-F07). They described needing to convey their shared understanding of the Hub’s work to clients and other services, particularly around the Hub’s limited role in treatment. Staff had a clear sense of what was required of them, although several noted that this often changed. All constructed similar value for the Hub’s work:

it’s invaluable…the majority of clients…if I hadn’t have made that referral they wouldn’t be in services (S02/009).

#### Participation: how engaged and committed were providers?

Key individuals with expertise in working with adults, CYPs, trauma, leading services and commissioning drove forward the implementation of the Hub. Representation of non-NHS organisations on the Hub steering group was praised but representation of service-users and the emergency services (themselves service users) was felt to be insufficient by some. Staff described feeling “honoured” (S02/F07) or “privileged” (S02/F10) to work at the Hub; one related that insufficient cover in their permanent role had negatively impact on their working and personal life. All felt that it appropriate for them to be at the Hub, and that the supportive atmosphere kept them engaged.

#### Collective action: did the change occur and who did what?

Hub project management was responsive to lessons learnt and changing needs. As a result, informing staff on part-time secondment of changes to processes could be difficult. Team members, typically specialised in either CYP or adult work, built confidence in each other and the all-age model of work by sharing knowledge and skills. With training, peer support, and frequently updated processes, staff generally felt they had the relevant skills, although some outlined unmet training needs in, for instance, dealing with the media. Most participants described the Hub’s interface with other NHS services as the most difficult aspect the Hub’s work, for example, having to grasp the processes and eligibility criteria for services across the UK:

we’d spend quite a lot of time in the early days…trawling through websites and ringing service after service to find out which was the most appropriate…it was a lot of leg work (S02/009).

Hub workers had to gain credibility with GPs and local services in order to progress clients’ referrals. All participants described arranging access to trauma-focused interventions as the most time-consuming part of the job, particularly for clients living outside of Manchester.

trying to help people access the support they need in a timely fashion, has been a big frustration…I think the sticking points are, it seems to be about the capacity within services that we refer to. (S02/F10).

Key barriers included inflexible pathways that would only accept self- or GP referrals. The widespread geographical reach of the client base highlighted the variable provision of specialist therapy across the UK. Local service capacity was sometimes limited or non-existent; waiting times often exceeded NICE guidelines. Access was particularly difficult for CYPs, as “there’s no standardised waiting time criteria to get children seen” (S02/F13).

#### Reflexive monitoring: what change occurred—why or why not?

Processes were refined through data collection exercises, such as case note audits, that staff reviewed together. Client surveys generally returned positive feedback, and staff recorded informal client feedback. Staff acknowledged that the trajectory of clinical outcomes was difficult to attribute to the Hub. Participants evaluated the Hub very positively. A consistent observation was the Hub’s responsive and evolving nature. Continual service reconfiguration was needed to respond to the changing needs of clients and to emergent limitations, such as introducing home visits in response to the limitations of using telephone assessments for people with complex needs. Hub staff began to see clients for therapy “when we realised that some of the services in the North West weren’t able to meet the timescales for treatment and that people really struggle.” (S02/F13).

## Discussion

This process evaluation expands upon and adds to the findings of the Kerslake Report. The findings are discussed below, with particular reference to their implications for actions and policy.

### Planning and resources

The Greater Manchester response was generally viewed positively, considering the Local Resilience Forum’s plans for major incidents did not include mental health support (p197, [35]). In line with the Kerslake Review, “Emergency plans for major incidents should incorporate comprehensive contingencies for the provision of mental health support” (p. 197, [35]). In Manchester, trauma-focused therapists and accredited supervisors were in short supply; we therefore add that emergency planning should include regular assessment of workforce capacity, the production of on-call rotas, and anticipatory training. Simulation exercises are essential to test local arrangements for co-ordination and delivery of the mental health response and address any identified gaps.

The financial impact on the local health economy of setting up the Hub is much bigger than areas would be able to absorb. There needs to be agreement between local and national commissioners and strategic leads as to how additional funding is identified in a timely manner to ensure appropriate resourcing of the mental health response.

### Data and information sharing

Considerable efforts were necessary to identify and approach those affected with offers of mental health support, although the Civil Contingencies Act (CCA) 2004 allows the suspension of normal data protection procedures and the sharing of individual identifiable data. “Responders have a duty to share information with partner organisations” (p15, [77]) and “should be robust in asserting their power to share personal data lawfully in emergency planning, response and recovery situations” (p8, [106]).

Pre-existing partnership and network arrangements enabled swift, research-based development of policy, messages and materials. Up to date materials should be made accessible by the NHS England EPPR team. Local government websites can be of variable quality as strategic communicative tools for the promotion of resilience [107], and the integrity of their information should be regularly assessed.

### Collaborative working

The basic seven-step model for designing and delivering the psychosocial response to a disaster (Table 2) [8] is unaffected by this evaluation, which reinforces the need to engage the right people at the right time. In this regard, the VCSE and statutory sectors have mutually corrective roles in providing routes for people in need to appropriate care (Table 2, Step 4).

Statutory sector leaders raised concerns about the inappropriate delivery of early therapy by some VCSE workers; the majority of VCSE leaders believed the health sector’s model resulted in unmet need in those whose symptom severity was below clinical thresholds for treatment. This predictable conflict over scope and status [68], was ameliorated in some parts of the response network through a culture of collaboration and close working relationships between sectors. The VCSE sector and the NHS outside of Greater Manchester remained less well integrated into the response network, despite efforts to improve information flow or referral quality and time.

The Home Office’s Victims of Terrorism Unit has been tasked with identifying and consolidating support pathways for those affected by terrorist attacks, and since the incident a VCS pathway has been developed for organisations including 3^rd^ Sector building on the work from Manchester and London.

### Leadership and workforce development

The development of the psychosocial and mental health response to the Manchester Arena attack has required leaders to communicate across organisational boundaries to deliver a shared vision working across agencies and systems. The Resilience Hub has provided an opportunity to develop trauma-based expertise within GM including the provision of training and resources. Staffing has initially relied on secondment of existing clinicians from across GM. Long-term sustainability of the Hub model will need to be considered, particularly on stepping down to local services. Trauma training and workforce development is required across community and specialist services in preparation for any future major incident.

Practical support for professionals and first responders should be integrated into response and recovery phases, and pathways developed to ensure that offers of support reach the people who may be affected. Cultural factors are likely to affect professionals in reporting mental health symptoms and engaging with mental health services. Subsequently, senior managers, HR and occupational health should consider formal and informal opportunities to support staff including; debriefs, drop-ins, Schwartz Rounds and support via primary care and community services so that services are sustainable over the long-term.

### Further research

Evaluation of the longitudinal trajectories of participants’ mental health responses to the Arena incident is planned, through a retrospective case series using individuals’ screening scores at multiple time points post-incident. Cohorts of individuals will be identified according to mental health trajectory, client group (CYPs, adults, and professionals), and time at which clients registered with the Hub. The Hub’s acceptability and economic impact will be assessed.

Taking the Kerslake Review’s findings on board, further research is needed to understand the range of individual reasons why some individuals had not received mental health report several months after the review and why some of those who did found the response unacceptable.

The sudden nature of contingencies makes researching the response to them difficult [108, 109]. However, employing national research infrastructure, studies can be prearranged and left in ‘hibernation’ pending an incident [110]. Researchers should plan research to understand how the materials and processes designed by the Resilience Hub can be implemented in a shorter time period, involving national authorities and research infrastructure organisations.

## Conclusions

All statutory sector and all but one third-sector interviewees considered the Resilience Hub a success given the absence of pre-event planning. Lessons, particularly regarding system development and integration, have been outlined, and implications for planning and policy explored. Any response to large-scale trauma must include an appropriately resourced mental health component embedded within the emergency response plan (EPRR), including consideration of support for professionals and first responders. The response should include all key stakeholders including local and national third sector agencies. The ability to transcend organisational and agency boundaries is crucial and requires leadership, collaborative working and an infrastructure to support data sharing and governance through pre-agreed arrangements. 

## Supplementary Information

**Additional file 1**. Consolidated criteria for reporting qualitative studies (COREQ) checklist.

**Additional file 2**. Interview guides.

**Additional file 3**. Sample quotes coded to each construct of the utilised conceptual/theoretical frameworks.

## Data Availability

Requests for further data not available in this publication can be directed to Daniel Hind, at the School of Health and Related Research. Email: d.hind@sheffield.ac.uk Tel: 0114 222 0707.

## Abbreviations

CAMHS: Children and Adolescent Mental Health Services
CBT: Cognitive behavioural therapy
CCG: Clinical Commissioning Group
CYP: Children and young people
EMDR: Eye movement desensitisation and reprocessing therapy
EPRR: Emergency preparedness response and recovery
GM: Greater Manchester
HSCP: Health and social care partnership, organisation formed as part of the integration of services
IAC: Inter-agency collaboration
IAPT: Improving access to psychological therapies
LID: Leadership in disasters
LRF: Local resilience forum
NHS: National Health Service
NPT: Normalisation process theory
PTSD: Post-traumatic stress disorder
RAG: Red-Amber-Green
SCN: Alliances of healthcare providers and commissioners which aim to improve quality and equity of care in priority service areas
VCSE: Voluntary, community and social enterprise
