# Supplementary material for: The psychosocial response to a terrorist attack at Manchester Arena, 2017: a process evaluation
Source: BMC Psychol. 2021 Feb 2;9:22. doi: 10.1186/s40359-021-00527-4 (PMC7852120; doi:10.1186/s40359-021-00527-4)
Supplement: Supplementary file 2 — Additional file 2. Interview guides. [file 40359_2021_527_MOESM2_ESM.pdf]

Assertive Out-Reach following The Manchester Arena Attack: Topic Guide

PART 1. PREAMBLE (10m)

Interviewee consent – discuss interviewee's consent to be interviewed:

- Check interviewee is happy to go ahead with the interview
- Go through consent form
- Check interviewee is happy for interview to be audio-recorded
- Ask if they have any questions or concerns before proceeding

The mental health response to the Manchester Arena bombing involved lots of organisations – from the third sector, education, the local authority and the NHS. We want to understand how that happened and whether there's anything that people elsewhere can learn from it. Especially we want to know What information do people need to respond to a disaster and how can it be collated and made available to the people who need it in a timely fashion?

PART 2. INTRODUCTION (5m)

Could I start with a little background on you? What is your role in your organisation and how long have you been in that role?

PART 3. BEFORE THE ARENA BOMBING

1. Had [you or your organisation] been involved with collaborative responses to other civil contingencies before the Arena bombing?

- 1996 Manchester bombing?

2. Before the arena bombing, were you consulted about involvement in the response to disasters?

- Were you part of the GM Local Resilience Forum, set up in response to the Civil Contingencies Act 2004?
- If yes:
    - How did that prepare you to respond to the Arena bombing?
- Were you involved in communication / information development?[1]
- Were you involved in the preparation of systems related to behavioural health impact and recovery?[1]
- Were you or your organisation involved in any training?[1]

PART 4. AFTER THE BOMBING

4. After the bombing, what discussions were a) you or b) your organisation involved with in developing the psychosocial response to the event?[1]

5. What are your views about the organisational and leadership structures that were in place to lead a response?[2]

What worked well, and why?

What were the challenges and barriers? Why were these challenging?

6. Were specific goals identified in the early days of the collaboration?

If yes- how were these goals developed, agreed and communicated to others?

What worked well and what were the challenges and barriers?

7. Was there a clear, shared sense of what to do (e.g. outreach, screening)?[2,3]

a) How were affected individuals/communities identified (e.g public and service personnel)?

b) How were decisions made and communicated to others?

c) What was the rationale for the offer that was developed .ie the different phases and the development of the central hub?

8. Was there appropriate expertise in developing and delivering the psychosocial response for both adults and young people?[2]

8. Was the response hampered in any way by lack of clarity over

a) funding mechanisms

b) data sharing arrangements?

9. What information was needed to organise the response?[2]

- Where was this information going to come from?
- Who was responsible for collating it and making it available?

What worked well and what were the challenges and barriers to obtaining the necessary information, and why?

10. Were you involved in mental health-related communications to service personnel or the public?[1]

- In the immediate response phase?
- In the recovery phase?

What worked well and what were the challenges and barriers to good communication, and why?

11. Were you involved in the clinical pathway development after the bombing?[1]

- In the immediate response phase?
- In the recovery phase?

What worked well and what were the challenges and barriers, and why?

11b Were you involved in the development of the resilience hub after the bombing?

- In the immediate response phase?
- In the recovery phase?

What worked well and what were the challenges and barriers, and why?

12. Were you involved in the development of training after the bombing?

- In the immediate response phase?
- In the recovery phase?

What worked well and what were the challenges and barriers to training, and why?

PART 5. EVALUATION BASED ON THE COLLABORATION THEORETICAL FRAMEWORK

13. How does inter-agency collaboration work in practice?

- Prompts: service fragmentation / boundaries, organizational complexity, planning horizons and cycles, funding mechanisms, values; interests.

14. Why did you think you needed to collaborate with other agencies after the bombing? [3]

15. What worked well, and what were the challenges and barriers to inter-agency collaboration, and why?

- Prompt: Was it difficult to collaborate?[3] Were there any barriers to inter-agency collaboration?[3] From their organisation in particular?
- Prompts: resources; loss of autonomy; collaborative culture; values; roles, boundaries (within organisation); sense of progress; sense of social purpose.

16. Have you been able to – have you wanted to – sustain collaboration? [3]

17. Could you rely on the people at the other organisations? [3]

18. As a champion for the resilience hub, were you supported by your organisation?

- Did you have to support your front-line staff?[3]

19. Was collaboration the right thing to do for your organistion?[3]

PART 6. LOGIC MODEL

"We want [their/your] guidance on whether the proposed logic model covers all the elements that are needed for the implementation and evaluation of a successful assertive outreach/ screen-and-treat programme".

That concludes the interview, thank you. Do you have any questions for me?

1. Flynn BW, Bushnell P, Lurie N. Leadership in Disasters. In: Ursano RJ, Fullerton CS, Weisaeth L, Raphael B, eds. *Textbook of Disaster Psychiatry. Second Edition*. Cambridge: Cambridge University Press; 2017:285-297.
2. Reifels L, Pietrantoni L, Prati G, et al. Lessons learned about psychosocial responses to disaster and mass trauma: An international perspective. *Eur J Psychotraumatol*. 2013;4(SUPPL.). doi:10.3402/ejpt.v4i0.22897.
3. Hudson B, Hardy B, Henwood M, Wistow G. In Pursuit of Inter-Agency Collaboration In The Public Sector. *Public Manag Rev*. 1999;1(2):235-260. doi:10.1080/14719039900000005.

Assertive Out-Reach following The Manchester Arena Attack: Topic Guide

PART 1. PREAMBLE (10m)

Interviewee consent – discuss interviewee's consent to be interviewed:

- Check interviewee is happy to go ahead with the interview

- Go through consent form (emphasise member-checking: **"there's a good chance you'll be identifiable to your colleagues, so we'll give you a chance to look over the transcript of your interview and remove or change anything you would rather we didn't include in the report"**)

- Check interviewee is happy for interview to be audio-recorded

- Ask if they have any questions or concerns before proceeding

The study objectives addressed by these interviews are:

- To describe the contexts in which the assertive outreach programme is delivered.

- To describe the implementation of the assertive outreach programme, including any barriers, facilitators, procedures and models you use to accomplish implementation.

PART 2. INTRODUCTION (10m)

- I would like to start with a little background on you: what is your role in the programme and how long have you been in that role?

- How did you get involved?

- Did you get any formal training?

- Has implementation gone as planned?
    - What challenges have there been?
    - What did you do when things went wrong? (change plan, stay the course)

- Who is manages you within the programme? What is their role?

- How often do they communicate with you (face-to-face?); what about?

PART 3. BRIEF COGNITIVE TASK ANALYSIS

We want to map out the steps in how you deliver your bit of the assertive outreach programme. Please can you think about what is involved; then, can you tell me about process in about 4-6 steps?

If we've got time at the end I'll ask you to walk me through how you've handled a typical case.

PART 4. NORMALISATION PROCESS THEORY

COHERENCE

Differentiation: Is this programme a new way of working?

Individual specification: Do you understand what the programme requires of you?

Communal specification: Does everyone involved agree about the purpose of the programme?

Internalisation: Does everyone involved grasp the potential benefits and value of the programme?

COGNITIVE PARTICIPATION

Enrolment: Do you believe the right people are driving forward implementation of the programme?

Initiation: Are they willing and able to engage others implementing it?

Activation: Can stakeholders identify what tasks and activities are required to sustain the intervention?

Legitimation: Do they believe it is appropriate for them to be involved in the intervention?

COLLECTIVE ACTION

Interactional workability: Does the intervention make it easier or harder to complete tasks?

Skill set workability: Do those implementing the intervention have the correct skills and training for the job?

Relational integration: Do those involved in the implementation have confidence in the new way of working?

Contextual integration: Do local and national resources and policies support the implementation?

REFLEXIVE MONITORING

Systematisation: How, if at all, are you able to judge how effective the programme is?

Individual appraisal: And, how effective do you judge the programme to be?

Communal appraisal: Have you come together with other people to talk about how effective it is?

Reconfiguration: Do you think it could be modified based on your evaluation and experience?

[If time, return to the cognitive task analysis]

That concludes the interview, thank you. Do you have any questions for me?
