# Supplementary material for: The psychosocial response to a terrorist attack at Manchester Arena, 2017: a process evaluation
Source: BMC Psychol. 2021 Feb 2;9:22. doi: 10.1186/s40359-021-00527-4 (PMC7852120; doi:10.1186/s40359-021-00527-4)
Supplement: Supplementary file 3 — Additional file 3. Sample quotes coded to each construct of the utilised conceptual/theoretical frameworks. [file 40359_2021_527_MOESM3_ESM.docx]

## Contents

[Codes from Flynn et al’s Mental Health Leadership in Disasters Framework 2](#_Toc529782852)

[Codes from Hudson et al’s Inter-Agency Collaboration Framework 4](#_Toc529782853)

[Codes from the Logic Model 6](#_Toc529782854)

[Codes from Normalisation Process Theory 10](#_Toc529782855)

Codes from Flynn et al’s Mental Health Leadership in Disasters Framework

| Code | Sample quote |
| --- | --- |
| Direct services to victims/ survivors | “we were trying to get hold of... names and addresses of everyone who’d gone there... difficult when people buy second hand tickets... we were being given some names directly via the blue light services... from the hospital part of the system... over several weeks afterwards... we actually wanted to contact everybody... in terms of the follow up and doing the triage... the difference [from Omagh] was the scale... we assumed there were more from Greater Manchester than subsequently turned out to be the case” (S03-F01) |
| Direct services to responders/ workers | “We had wanted organisations to give us the contact details of first responders so that the hub could contact them directly to engage in the screening process... none of the provider organisations had been happy to do that... that whether that really hampered the response to professionals... for different reasons like Occy health departments, HR departments...” (S02-F02) |
| Consultation to leadership | “There was quite a broad group of us at first and they were bringing really as many people as they could think of... there was some communication by email but there were fairly regular meetings ... then a sort of reduction to a core group of people for the sort of longer term governance ... it seems to me to have been fairly pragmatic in terms of the people who need to just get on with... I felt I’ve been involved the appropriate amount for what my influence should be at either end of it really” (S07/F01) |
| Communication/information development & dissemination | “A lot of the people ... were involved in media... or communication ... particularly for children...we started to encounter some of the normal barriers ... like sharing information... and had to start thinking about how we could communicate with people sensitively... and use that information without people feeling that their confidentiality had been breached.... the outreach approach was as I said we’d categorised people and, and were very clear that there was going to be a group of people who was struggling in silence really and that we needed to reach out to. And, actually, even just that connection of saying “We’re thinking about you, how are you doing?” (yep) even if it’s through email and letter, actually people really value that... the media were at times not being very helpful” (S02/F03) |
| Evaluation | the sense of what to do was to, um, support what was needed ... for the short term... and then to put something in place... which included evaluation… this is taking us into a new place... the last time something... equivalent had happened was the... London tube attacks, and there’d been an enormous amount of learning that came from that... how could we make sure that whatever we learned from this that we played straight back into future planning (S09/F01) |
| Systems development & integration | “a regular part of that... fortnightly conversation was ... what does the pressure feel like at the moment both from the you know the acute trusts and from the mental health trusts,and what actions do we need to take and then use the contacts on that call to drive the actions out through specific networks... We’d looked to cover the network that needed communicating with through that fortnightly call... the core premise of the service... was... identifying those that needed support and referral into services, where we started to pick up where people were hitting barriers in terms of access to services, then we set up a kind of an escalation protocol... if people were experiencing... unacceptable delays in service... [then we] would use the NHS England network” (S03/F02) |
| Training/ education | “through health education England we did an audit of who was qualified... what therapies and where they worked... then also arranged for some additional training... in CBT and EMDR... We... collared them on the day... said... “What are your skills?”, “Where do you work?” and “Would you be willing to help?” ... it’s fine identifying need, isn’t it? But if you don’t have a workforce that’s fit to respond...” (S02/F03) |
| Policy development and implementation | “The universal public health offer [was] meant for the general public... The targeted offer was for people who were exposed to the trauma of the event... delivered by various providers across Greater Manchester and wider... and it was monitoring how people were, screening for high risk groups. The idea of the hub was there needed to be a point of contact for people... there was a message consistently going out that there was a group of people who could help you manage that response.” (S02-F06) |
| Research | “I had spoken ... to my colleagues who had responded to Tunisia and Paris and asked them... ‘I’ve read all your papers but is there anything you... you would do differently?’ and unanimously they all said they’d respond... and they’d contact victims quicker.” (S02/F01) |
| Advocate for science and evidence | “there were lots of people willing to offer help not a lot of which was always evidence based... I went to a meeting... to ...bring together voluntary sector groups and ... ...every time we sat at a table with this particular group they got up and left, so they wouldn’t be challenged by us....some of those people subsequently have come to the hub and been quite damaged by what they were offered...we were giving normalisation messages but perhaps we weren’t giving enough support to say what you also could do, so that gap it was filled by people saying oh come here you can have counselling from us” (S02-F06) |

Codes from Hudson et al’s Inter-Agency Collaboration Framework

| Code | Sample quote |
| --- | --- |
| 01. Contextual factors - expectations and constraints | “the whole justification for the GM HSCP is... we’re breaking down... barriers between... organisations... it’s built on many, many years where people have got used to that...” (S03-F01) |
| 02. Recognition of the need to collaborate. | “there’s... inevitably some competition... some... sense of... whose view is best when there are differing views... there was... a little bit of... tension between providers... about who would host it ... some difficult... conversations about why particular people felt that they were in a better position which wasn’t necessarily asset-based but was more around how these organisations normally conduct themselves and do business... so there was a bit of reverting to type... reverting to the normal way of things... that was brokered... by the partnership and by the commissioners” (S02/F03) |
| 03. Identification of a legitimate basis for collaboration. | “because of the enormity of the event, human relationships went across organisational boundaries in the first few days... it didn’t matter who you work for... people just came together in that immediate aftermath... positivity, in the sense of ‘we need to get the job sorted here’ lasted for quite a while... the awfulness of the incident... it helped people transcend organisations...” (S01/F02) |
| 04. Assessment of collaborative capacity | “what was absolutely, incredibly positive about it was there was a willingness to move across GM to support this and our relationships with our colleagues in other trusts, so we were able to really mobilise things across both Acute and Mental Health Trusts... I've never in all the years I’ve been in the NHS... seen people ... shelve that and say let’s just get this response up and running, let’s just put aside any 'I’m from this trust, you’re from that trust, who commissions this, who doesn’t'. There was a real willingness to really make this work really quickly... lots of us would have known each other but not necessarily known all of the, the stuff that other people did. So there was that learning curve of saying oh yes, actually, that person has particular expertise around this. Um, so you have to put together a group and that sort of developmental phase in that group.” (S02-F06) |
| 05. Articulation of a clear sense of collaborative purpose | “the main goal... we all agreed... was... minimising long-term... psychological difficulties... that was communicated through ... meetings, papers ... "this is the raison d'être of the psychological support through the Hub… how we achieve it... was... flexible...” (S01/F01) |
| 06. Building up trust from principled conduct | "My [counterpart] in [other organisation] and I, happen to get on very well and we... are both of the same mind around get the function sorted rather than the form. That helps. I think if you’ve got leadership that says, ‘you know what, it doesn’t really matter, let’s just crack on and do it’, that, that makes the difference." (S05-F06) |
| 07. Ensuring wide organizational ownership | It was a system wide response even to the clinical pathways group. So when I say that I mean health social care, voluntary, community sector, it was social care. It was colleagues representative of all of those sectors to work together on the clinical pathway offer.... (S04-F02) |
| 08. Nurturing fragile relationships | when you look at Manchester ... that there was already a high level of trust, so people knew, people had already been tested in terms of the relationships... part of planning is making sure that you maintain those relationships ... if you find an area ... of weakness ... you need to ... use your own systems ... to ... try and overcome it. (S09/F01) |
| 09. Selection of an appropriate collaborative relationship | “We wanted a co-ordinated offer - using resources across the system.” [coalitional or federative working in the response phase] “we think the model needs to be a centralised hub model. Because that will allow us to ... maximise ... the expertise in the system and get it more cost effectively” [unitary model in the recovery phase] (S02-F02) |
| 10. Selection of a pathway | “If I can get people to cooperate why would I waste my time ... [as]happened in London where people went through a convoluted procurement programme” [the rejection of market mechanisms] (S03-F01)  “there is a single... Senior Responsible Officer for the GM Mental Health Strategy …[and a] Lead Commissioner so it was pretty easy for us to have that authority to pull people together... we’d have a direct link in to the main response system coming together at the higher than gold command really and through to Central Government.” [use of hierarchy] (S03-F01)  “there were already networks in place with colleagues in the voluntary sector, with colleagues in the Education sector, so what I observed was the on-the-ground relationships were very strong and people were very quickly mobilizing those networks and relationships.” [The use of networks] (S09/F01) |

Codes from the Logic Model

| Code | Sample quote |
| --- | --- |
| #1. Develop and integrate systems | we knew that our job would be to identify the people who needed help and make sure that their local NHS services or other relevant services were able to deliver that help and be able to... help people navigate through the mental health system ... we told the whole system that we were setting up... and if there was anyone that they thought we could help... they just needed to get their consent and... pass their details on (S02/F04) |
| #2. Pre-event training  (not conducted) | you should have had a planning process that would have identified safe… practitioners and have a kind of reserve group of people that you could call into action following a serious ... untoward event... (S02-F06) |
| #3. Establish leadership relationships | on the morning of the bombing I contacted NHS England directly.. to discuss what had happened and we agreed that... they needed to link into a regional core... I emailed the GM clinical leads... the health and social care partnership or the strategical clinical network... pulled together a teleconference... lunchtime [the day after the bombing]...they managed to pull together... key people on that call very quickly (S02-F02) |
| #4. Build communications team and strategy | after the first 12 hours there were twice daily calls from the communications aspect perspective (S01/F02) |
| #5. Assemble response team. | we did a... trawl at the start to see how many MDR therapists .... trauma focussed CBT therapists .... family therapists... with hindsight, there would be something really important about having a regular update, in anticipation of major incidents of where you’ve got capacity and how you can draw that in quickly....knowing what capacity we’ve got in the system to deal with it, and also who manages that capacity. And how can it be freed, whilst also ensuring that your core business happens day to day because there was a backlash, minor, but there were some people who felt that this was this was taking staff away from basic core business that we also had to do.in the North West, we have what we call the Psychological Professionals Network... I spoke to the chair of that, who’s a colleague of mine in... about ... if I needed to put something out through that group ... that that needs to again be built in, and that workforce dynamic needs to be built into the operational management plan for major incident, is to have that sort of sitting, sitting ready to lift and load. How many staff do you train in NDR, how many staff do you train in trauma focused work etc. (Yeah) And you need someone who can access that information pretty quickly....(S02-F06) |
| #6. Identify intervention efficacy data and tailor intervention to situation.  Prepare for mental health impact of work | we’d immediately started to research... incident that occurred in Nice... London ... the Tunisia attack in 2015... people involved... up to even 2017 had not had any sort of support... the lack of coordination in that aftermath of the attack... tipped it into... 'there’s... a lot of people... from other areas... how do we develop that in a more coordinated way?’ ... (S01/F02) |
| #7. Evaluation and research functions built into systems | we had very early conversations around wanting to put consent and research on our website as people were doing the screenings was trying to get people to opt in. Because we knew that, we would want to be using data and to be thinking about research and evaluation (S02-F02) |
| #8. Wider network of cross-sector leaders (social capital, recognising social dimensions and sources of resilience) | we wanted to think about how we used the resources across the full system in a more coordinated way...we didn’t... have a sense of all the... voluntary sector organisations, so... there was quite a lot of work trying to understand who we needed to be tapping into... The ability to communicate with the wider system... multiple CCG’s, providers... really needs to be thought through... (S02-F02) |
| #9. Establish centralised register identifying all survivors and those affected by the incident | "we were trying to get hold of... names and addresses of everyone who’d gone there... difficult when people buy second hand tickets... we were being given some names directly via the blue light services... from the hospital part of the system... over several weeks afterwards... we actually wanted to contact everybody... in terms of the follow up and doing the triage... the difference [from Omagh] was the scale... we assumed there were more from Greater Manchester than subsequently turned out to be the case" (S03-F01) |
| #10. Social media systems and outreach materials (e.g. leaflets) | I was liaising with the GM gold command to produce evidence based age appropriate materials for adult, children and staff.... the universal offer... there was a message consistently going out that there was a group of people who could help you manage that response, whether it was that you needed to know our services were aligned, pathways, or whether you were an individual affected by the event. (S02-F06) |
| #11. Online screening systems, and patient records | we got the special version of PCMIS system set up... with a portal that people could refer into and we’ve had all screening questionnaires put on... working with the clinicians to work out what the pathways were and ... trying to ... make that happen through the electronic system....in the background... for various questionnaires some for children and young people and some for adults and there’s a different scoring mechanism... then depending on the scores... you get a different auto-response... and then you come in to different holding lists within the system. (S02/F04) |
| #12. Telephone, online messaging and email systems used to deliver support | “[Name] really kind of did a huge amount of work on that so you know helped to locate premises where they were located, they actually found somewhere that was right next to their Trust Headquarters which already had all of the cabling in terms of NHS computer stuff fed into that building.” (S01/F01). |
| #13. Administrative, recovery and clinical workers | some future learning would be to have some agreements in principle ahead of anything as to where the resource would come from and how many staff the full-time equivalent of people might contribute (S02/F01) |
| #14. Training materials | we had a couple of workshops in August [2017]... one for CBT Trauma Therapists and one for EMDR Trauma Therapists... [with] external national speakers... they were oversubscribed so we recorded them ... [and asked] the Psychological Professions Network... to host that material on a password protected webpage" (S02/F01) |
| #15. Track operation / integration of systems | situation report... but it was always shorthened to 'sit rep' ... the various kind of things that you have in the first days of kind of silver command and gold command... sit rep calls and we used to have them once a week and everybody... lots and lots of different people were dialling [in]... about progress and where we’re up to... (S02/F04) |
| #16. Leadership consultation | The GM HSCP... incorporates the functions of NHS England for GM, and NHS England... is a Category A responder in the civil contingencies legislation... the morning after... the bombing ... [As part of Gold Command, the Chief Officer of the GM HSCP]... asked [Name] to start thinking about a mental health response as part of recovery... the afternoon of the 23rd... [Name] just really sought to get as many people from the, from the, from the system across the mental health trusts and other relevant parties joining a conversation. (S03/F02) |
| #17. Assertive outreach to contact all survivors | “It was done by essentially finding networks and routes to them, so I suppose I’m one of them routes... I was the outreach to particularly the 16-19 year olds” (S07/F01) |
| #18. Mental health screening | When we started we made an active decision to send out screening to, and make contact with this Yammer group... they were our first cohort, so we kind of specifically targeted those and then widened it (mmm) and I think it was August when we did our first big, generic um, you know going out to screening. August/September. (S02/F03) |
| #19. Train/ educate / monitor staff welfare | "the programme lead for... Salford Cognitive Therapy Centre... organised a programme, contacted the speakers etc... bespoke psychological training around managing trauma for adults and children... and got some really incredible speakers ... with real national expertise and international expertise" (S01/F01) |
| #20. Process evaluation | “This evaluation actually highlights the need for that to be done.” (S04-F03) |
| #21. Universal interventions offered to everyone affected with information about the Resilience Hub | Yeah, well the universal offer I would say, was more to the, if you think of it, if you construct it on a sort of public health offer, that was meant for the general public, so that was for anybody, anybody in Manchester was affected by this. (Yeah) Well actually lots of people outside of Manchester were affected by this, so we were pleased that we got, you know there was some information on Blue Peter for kids |
| #22. People affected by the attack complete mental health screening | “with regards to the screening, the professionals that we have tried to contact, that has been the least receptive. … very, very poor uptake. And that sort of does worry me, that you know, people who saw these children first hand in those early hours of the day, you know the, and or others, whether it was the Arena staff, the ambulance, it doesn’t matter who it was you know the taxi drivers, erm, they’re all professionals in their own erm, in their, in their own way aren’t they? (I-yes). You know, have we got, are we, have we got something brewing that we’re just not prepared for? (I-yeah) In that group, yeah.” (S04-F01) |
| #23. Respondents triaged according to established clinical cut-off point | “How do we know who are the right people to look at that minute?” And “Who’s safe to leave?” … if they rated themselves as risky we … put a trigger into the system to identify that. anybody who didn’t score clinically significantly umm on the rating scales we didn’t make contact with but we sent them a letter (yeah) saying “Thank you for your response, you’re obviously doing really well” you know, “You’re using strategies, here’s some further information. Contact us if you want to” but we felt we couldn’t do that with children… (S02-F03) |
| #24. Those who need it have phone/email support from the Hub | “Sometimes, we say, “well, actually, I think, this organisation would be great and we have another conversation and say well would you like us to pass your details onto them?”, you know, but we then have to get the consent and then we, you know, so we consent them and we send them on, but we are more likely to say to people...” (S02-F04) |
| #25. Those who need it signposted/referred to primary care or specialist interventions | “and it’s not dissimilar from people where we say to them would you like us to make a referral for you to this IAPT service cause with the IAPT services they’ll only take self-referral so we can say to people would you like.. this is we’ll look it up for them, here’s the link if you want to go and self-refer or we’ll say you know would you like us to, if you’ve got time on the phone, but we’re just gonna need to ask you a loads of questions so that we’ll fill it in for you now on the phone if you want.” (S02-F04) |
| #26. Barriers to care access addressed proactively | where we started to pick up where people were hitting barriers in terms of access to services, then we set up a kind of an escalation protocol... if people were experiencing... unacceptable delays in service... [then we] would use the NHS England network (S03/F02) |
| #27. Staff address emotional aspects of work | We’re part of the group with the Point of Care Foundation that trains some facility leaders to be able to offer Schwartz Rounds. But, again, it’s finding a home so that we get staff released. And we know that – staff it’s gonna be a slower burn with them. It may be up to 3 to 5 years before we get some of the major issues coming through for staff, but actually trying to do some early intervention around that is such a critical thing, and we think Schwartz Rounds could be a good vehicle for that to at least get people to start to recognise that maybe they haven’t dealt with it particularly well and that actually its ok to say that they haven’t. (S02-F06) |
| #28. Clinical evaluation | No quotes. Clinical evaluation ongoing at time of writing. |
| #29. Prevention of distress | No quotes. Clinical evaluation ongoing at time of writing. |
| #30. Reduction in distress | No quotes. Clinical evaluation ongoing at time of writing. |
| #31. Fewer people develop severe distress | No quotes. Clinical evaluation ongoing at time of writing. |
| #32. Reduced likelihood of people developing severe difficulties consistent with psychiatric diagnosis | No quotes. Clinical evaluation ongoing at time of writing. |
| #33. Staff mental health preserved | No quotes. |

Codes from Normalisation Process Theory

| Code | Sample quote |
| --- | --- |
| **1. Coherence** |  |
| 1.1 Differentiation | “it’s an outreach service, its psychologically-led, and it’s all-age...It’s definitely a new model for me and I think it’s also a new model for GM and I think, I would say probably it is, a completely innovative model throughout…certainly the NHS.” (S02/F12) |
| 1.2 Communal specification | “I think that’s one of the refreshing things about actually working at the hub, is that people have a kind of er, shared goal I guess and I think people at the hub are particularly committed to try and make it work as best they can... It’s to respond to individuals in a sensitive way as effectively as possible, to assess as quickly as possible what the support needs are and try and help them access er, the right kind of therapy to meet their needs.” (S02/F13)  “The lack of clarity for people external to the hub because it’s so new...it’s taken people, I think, a long time to realise that we don’t actually deliver therapy, that we’re there to help people access therapy” (S02/F08) |
| 1.3 Individual specification | “from the very beginning…it’s been made very clear and with lots of support about...what people want me to do... as the hub evolves, then…I believe that my role evolves...it’s an evolving thing and I evolve with it and...that’s what I expect.” (S02/F10) |
| 1.4 Internalisation | “I think some, some of the value for me has been about showing that it is possible to do things differently...it had to be a collaboration between four different trusts...it’s made me think ‘are there other...ways that mental health services could be organised differently that could be better?’...being able to work together across child and adult mental health, across four different mental health providers erm, supporting people who live across the UK I think, that’s the thing more than anything, I think.” (S02/F12) |
| **2. Cognitive participation** |  |
| 2.1 Initiation | “what has been really helpful is the wide circle of engagement they have... with VCSE sector being around the table with commissioners ... Having both CYP and adult clinical leads and deputies ... I think as well having a service lead who previously been a commissioner also helped... it would have been useful to maybe have an OD lead from one of the blue light services or an occy health person” (S02/F07) |
| 2.2 Enrolment | “I was really pleased to do it...one of the thing that I feel very strongly about...is that we need to get certainly psychologists, umm, mental health practitioners etc. out of health and into supporting the wider system as well, and getting that psychological thinking out into the wider system and I think that’s really important” (S02/F08) |
| 2.3 Legitimation | “we do need people who are you know on the front line ringing clients, outreaching erm everyday cos without that they would be a, a handful, not even a handful, majority of clients wouldn’t be in services or wouldn’t feel supported” (S02/F09) |
| 2.4 Activation | “I think there’s had to be a lot of commitment from the provider organisations...I think there had to be some acknowledgement that [they] had to be quite flexible...I think there’s something about a staff team that was able to be really reflective and consider its own processing...I think there’s a real will to change and adapt and I think that’s important as well...to not become too invested in a particular way of doing things and I think that’s really evident in the hub” (S02/F08) |
| **3. Collective action** |  |
| 3.1 Interactional workability | “So there was...very much an agile project plan...the learning from week to week is built in to the next week's planning and objectives...ensuring that the processes were being followed by our clinicians, recovery workers and our administration staff...the nitty gritty of ok so if we change this process it’s going to take X amount of time for that to be communicated out to all of our staff” (S02/F07) |
| 3.2 Relational integration | “the whole system was incredibly supportive of one another” (S01/F01)  “**Interviewer**: ...do you have confidence in the Hub's way of working? And the...others at the Hub? **Interviewee**: ...absolutely, without hesitation.” (S02/F12) |
| 3.3 Skill set workability | “I felt that I had the skill set to do that and felt very supported to be able to do that as well... we have tried to be consistent so a family will have a single point of contact but... that means we’re asking our CYP clinicians to be able to support parents and for our adult clinicians to be able to support children and I, I have no idea how you could confidently do that in other services” (S02/F07)  “I definitely got the right training at the time, there’s a lot of support” (S02/F09) |
| 3.4 Contextual integration | “there are different services have different referral criteria and different waiting lists...trying to help people access the support they need in a timely fashion, has been a big frustration...I think the sticking points are, it seems to be about the capacity within services that we refer to. So that’s a commissioning thing, I suppose” (S02/F10) |
| **4. Reflexive monitoring** |  |
| 4.1 Systematisation | “recently we’ve done a snap survey and sent that out erm which we’ve had a lot of positive feedback from...at other points we’ve completed audits so that looks at it basically went through everybody, case by case” (S02/F09) |
| 4.2 Communal appraisal | “informally...all of our senior management team, all our discussions, all of our papers are all around, you know how can we make this as effective as possible.” (S02/F12)  “I’d say they really encouraged ideas ... to be coming from anyone within the team... what people felt like they’ve done well in or where there were gaps in ... the service that we are providing at team meetings... every staff member had an opportunity to come to at least one and then there was written minutes and feedback shared” (S02/F07) |
| 4.3 Individual appraisal | “I think it’s very effective and it appears to be incredibly effective, but one of the things, the feedback that I do remember reading was that um, it’s only as effective as what it enables somebody to do...So, we can...find services, we can get somebody into a service...but then there might be a huge, long waiting list and then it’s not effective because that person is sitting and waiting” (S02/F10) |
| 4.4 Reconfiguration | “in a sense it feels like...it is always changing...I suppose one of the things is that we are on our next round of secondments and erm, you know there is a, certainly a group of young people and erm adults that we are having more contact with now who are more complex...and risky, so that has changed a bit...so we are going to be looking for people who’ve got experience of risk management....we need to be fluid to meet the needs of, of our population.” (S02/F12) |
